# Identification and Characterization of a Ste20-Like Kinase in *Artemia* and Its Role in the Developmental Regulation and Resistance to Environmental Stress

**DOI:** 10.1371/journal.pone.0092234

**Published:** 2014-03-17

**Authors:** Rong Zhou, Yu-Xia Sun, Wei-Jun Yang, Fan Yang

**Affiliations:** Key Laboratory of Conservation Biology for Endangered Wildlife of the Ministry of Education and College of Life Sciences, Zhejiang University, Hangzhou, Zhejiang, People's Republic of China; Louisiana State University Health Sciences Center, United States of America

## Abstract

**Background:**

To adapt to extreme environments, the crustacean *Artemia* has evolved two alternative reproductive pathways. During ovoviviparous (direct) development, nauplius larvae are produced. In contrast, *Artemia* females release encysted diapause embryos (cysts) via the oviparous pathway. To date, the cellular mechanisms that regulate stress resistance of *Artemia* remain largely unknown. Ste20-like kinase (SLK) participates in multiple biological processes, including stress responses, apoptosis, and cell cycle progression.

**Principal Finding:**

We isolated and characterized a member of the SLK superfamily termed ArSLK from *Artemia parthenogenetica*. The *ArSLK* gene is transcribed throughout both ovoviviparous and oviparous development; however, the protein is located mainly in the nuclei of stress-resistant diapause cysts, unlike the nauplii and nauplius-destined embryos where it is cytoplasmic. Interestingly, exposure of nauplii to heat shock, acidic pH, and UV irradiation induced the translocation of ArSLK from cytoplasm to nucleus. This translocation was reversed following stress removal. Moreover, under physiologically-stressful conditions, the nauplius larvae produced by adults after gene knockdown of endogenous *ArSLK* by RNAi, lost the ability of free-swimming much earlier than those of control larvae from females injected with *GFP* dsRNA.

**Conclusions/Significance:**

Taken together, this study demonstrated that trafficking of ArSLK between the cytoplasm and the nucleus participates in regulating the stress resistance of *Artemia*. Our findings may provide significant insight into the functions of members of the SLK superfamily.

## Introduction

The brine shrimp *Artemia parthenogenetica* is widely distributed in hypersaline environments characterized by such things as anoxia, high salinity, pH extremes, high UV irradiation and temperature extremes. This primitive crustacean has evolved two independent reproductive pathways that enable it to adapt to these environments. Under certain conditions females produce and release nauplius larvae (the ovoviviparous pathway) [1], [2]. By contrast, under harsh conditions *Artemia* releases encysted diapause embryos (cysts) in which development is arrested and metabolic activity is greatly depressed (the oviparous pathway) [1], [2]. The striking stress resistance of *Artemia* makes it an ideal model system in which to study the adaptive mechanisms involved.

The accumulation of molecular chaperones in high levels has been implicated as one of these mechanisms in diapause embryos. Among these molecular chaperones, p26 and artemin have been well characterized. The small heat shock protein, p26, located in the nucleus and cytoplasm of diapause embryos, functions as a chaperone for proteins [2]–[6], while the ferritin-like protein artemin, located only in the cytoplasm, exhibits chaperone activity for proteins as well as RNA [7], [8]. Mammalian cells expressing p26 or artemin display enhanced tolerance to heat stress [6], [9] and those expressing p26 are also more resistant to desiccation [10]. Another abundant molecule is the disaccharide trehalose, which reaches about 15% of the diapause embryo's dry weight, has been shown to be critical to desiccation tolerance [2], [11]. Although these results represent progress in our understanding of stress tolerance in *Artemia*, the detailed regulatory mechanisms involved in this resistance remain to be described.

Sterile 20 (Ste20) kinase, of much significance in our study, was initially identified as a mitogen-activated protein kinase kinase kinase kinase (also known as MAP4K) involved in the mating pathway of the yeast *Saccharomyces cerevisiae* [12], [13]. Mammalian homologs of Ste20 kinase have since been identified and are classified into the p21-activated kinase and germinal center kinase subfamilies on the basis of the distinct locations of their kinase domains [14].

Ste20-like kinase (SLK) belongs to the germinal center kinase subfamily, containing a conserved N-terminal kinase domain and a variable C-terminal regulatory domain [14]. This protein kinase interacts with specific partners and participates in various signalling pathways, including those involved with the control of programmed cell death, cell proliferation and cell migration [15], [16]. Overexpression of SLK induces apoptosis, which is mediated by its ability to activate #c-Jun N-terminal kinase# and p38 MAPK kinase activity [17], [18]. Studies on *Xenopus laevis* oocytes and human somatic cells revealed that SLK activity is also required for the cell cycle transition from G2 to M phase [19], [20].

In this study, we examined the role of SLK (ArSLK) in the stress resistance of *Artemia*. We found that accumulation of ArSLK protein in the nucleus of diapause cysts suggests that it might play an important role in developmental arrest to resistant stress. Furthermore, gene knockdown of *ArSLK* verified that a decrease in the amount of ArSLK reduced the degree of stress resistance. The results to be presented here indicate that the nuclear translocation of ArSLK is involved in *Artemia* stress-resistance and sheds further light on additional functions of the SLK superfamily.

## Materials and Methods

### Animals

*Artemia parthenogenetica* cysts from Gahai were a kind gift from Prof. Feng-Qi Liu of Nankai University, Tianjin, China. Animals were raised in artificial sea water (Blue Starfish, Hangzhou, Zhejiang, China) at 25°C and were fed once every 2 days with *Chlorella* powder. Ovoviviparous *Artemia* adults, releasing nauplius larvae, were raised in 55 g/L artificial seawater under a regime of 16 h light and 8 h dark, while oviparous adults, releasing diapause cysts, were incubated in 110 g/L artificial seawater under a regime of 4 h light and 20 h dark. In both reproductive pathways, early oocytes (EO) are formed in the paired ovaries, and then mature in the oviducts to the late oocyte stage (LO), followed by passage into the ovisac (uterus) where they become early embryos (EE) and then later embryo stages (LE) that will give rise either to nauplius larvae or to diapause cysts, both being released into the environment. Specimens were collected at each developmental stage as described above.

Diapause cysts (Dp) were gathered, and then frozen at −20°C for 3 months to terminate diapause and produce post-diapause (activated) cysts (Pd). For hatching, post-diapause cysts were hydrated at 4°C for 5 h and then incubated in 28 g/L artificial seawater at 25°C with continuous light. Specimens were collected after 4, 8, 12, 16, 20, 36 and 48 h of incubation.

### Molecular cloning of ArSLK cDNA

To obtain a partial *ArSLK* cDNA fragment, 1 μg of total RNA from diapause cysts was reverse transcribed using oligo (dT) primers and MMLV Reverse Transcriptase (TaKaRa, Shiga, Japan) in a 10 μl reaction. Two rounds of PCR were then performed using two pairs of degenerative primers (ArSLK–F1 and ArSLK–R1, ArSLK–F2 and ArSLK–R2) (Table 1). A 460 bp fragment was obtained and a BLAST search of the deduced amino acid sequence was performed. To obtain the full-length *ArSLK* cDNA sequence, 3′ and 5′ rapid amplification of cDNA ends (RACE) reactions were performed using gene-specific primers (ArSLK 3′F1, ArSLK–3′F2, ArSLK–3′F3, and ArSLK–3′F4 for 3′ RACE; ArSLK–5′R1 and ArSLK–5′R2 for 5′ RACE) (Table 1) and the FirstChoice™ RLM-RACE kit (Ambion, Grand Island, NY, USA). The 5′ RACE reaction produced a fragment of 305 bp. The first round 3′ RACE reaction, performed using the ArSLK–3′F1 and ArSLK–3′F2 primers, produced a 1.5 kb fragment containing a pseudo poly (A) tail. The second round 3′ RACE reaction, performed using the ArSLK–3′F3 and ArSLK–3′F4 primers, produced a 2.5 kb fragment. To verify the full-length ArSLK cDNA sequence, a pair of gene-specific primers (ArSLK–F and ArSLK–R) (Table 1) was used to amplify a 4020 bp open reading frame fragment. The PCR product was subcloned into the pUCm-T vector (Sangon, Shanghai, China) and then sequenced. The nucleotide sequence of this ArSLK-encoding cDNA was submitted to GenBank and the accession number was KC818632.

**Table 1:** Nucleotide sequences and positions of primers used in polymerase chain reactions.

Primer	Length (bp)	Position	Direction	Sequence (5′-3′)
**ArSLK-F1**	23	253–275	F	GCNTTYGGNAARGTNTAYAARGC
**ArSLK-F2**	23	244–266	F	GGNGAYGGNGCNTTYGGNAARGT
**ArSLK-R1**	23	691–713	R	ATCCARTANGGNGTNCCDATRAA
**ArSLK-R2**	23	700–722	R	TCNGGNGCCATCCARTANGGNGT
**ArSLK-5′R1**	21	636–656	R	GCAGACACACCAAAATCCGCT
**ArSLK-5′R2**	21	285–305	R	GCAGCTAATGCTTTTGTCTCG
**ArSLK-3′F1**	21	595–615	F	GCTGGAAATGTTCTACTGACC
**ArSLK-3′F2**	21	664–684	F	AAGTCCACACTTCAGAAACGA
**ArSLK-3′F3**	20	1760–1779	F	TTATGCTTGGCTATGAACCT
**ArSLK-3′F4**	20	1981–2000	F	GGCAATGGCATAGACAAAGC
**ArSLK-F**	22	109–130	F	ATGTCATTTTTCTCAAGGAAAG
**ArSLK-R**	23	4106–4128	R	TTACGAATTCGACGATATTGACG
**ArSLK-RTR**	20	2128–2147	R	TCCTCAAGTTTGCTATCTGC
**Tubulin-F**	20	446–465	F	GCAGTGGTCTACAAGGTTTC
**Tubulin-R**	22	774–795	R	ATCAAAACGAAGGCTGGCGGTG
**ArSLK-dsF**	29	1160–1179	F	GCTCTAGACGGTTCCCTCACCACCTACA
**ArSLK-dsR**	28	1720–1739	R	GGAATTCTGCCCGACGTGGTCAATTAC

F and R indicate the forward and reverse directions, respectively.

### Immunoblotting

Proteins extracted from tissues of *Artemia* at different developmental stages were prepared using TRIzol (Invitrogen, Carlsbad, CA, USA) reagent, as per the manufacturer's instructions, and then quantified by the Bradford method [21]. Forty micrograms of each total protein sample were separated on 10% SDS-PAGE gels and then transferred to PVDF membranes (Roche, Indianapolis, IN, USA). After incubation of membranes with primary antibodies at 4°C overnight, specific proteins were detected using the BM Chemiluminescence Western Blotting Kit (Roche, Indianapolis, IN, USA). To obtain the anti-ArSLK antibody (HuaAn, Hangzhou, Zhejiang, China), rabbits were immunised with a peptide that was based on amino acids 391−405 of ArSLK. The anti-α-tubulin antibody (Sigma, St. Louis, MO, USA) was purchased.

### Cell fractionation

Cell fractionation was performed as previously described [22]. In brief, specimens were homogenized in a Dounce homogenizer on ice in 4-(2-hydroxyethyl)-1-piperazineethanesulfonic acid (HEPES) buffer (10 mM HEPES, 4 mM MgCl_2_, 1 mM phenylmethylsulfonyl fluoride (PMSF), pH 7.5). To obtain the cytoplasmic fraction, homogenates were centrifuged at 3300 g for 15 min at 4°C and the supernatant was retained. To obtain the nuclear fraction, the pellet was re-suspended and re-homogenized in fractionation buffer (10 mM Tris, 10 mM NaCl, 10 mM EDTA, 0.5 mM EGTA, 4 mM MgCl_2_, pH 7.4), loaded onto 45% (w/v) sucrose buffer drop by drop, and then centrifuged at 13000 g for 15 min at 4°C. The pellets were washed once and then dissolved in EBC buffer (50 mM Tris, 120 mM MgCl_2_, 1 mM EDTA, 0.5% NP-40, 1 mM PMSF, pH 7.5). Proteins were quantified by the Bradford method [21]. Equal amounts of protein were loaded for immunoblotting. Anti-α-tubulin (1∶5000) (Sigma, St. Louis, MO, USA), anti-histone H3 (1∶5000) (Epitomics, Burlingame, CA, USA), and anti-ArSLK (1∶1000) (HuaAn, Hangzhou, Zhejiang, China) antibodies were used for detection.

### Real time PCR

Specimens from different developmental stages were snap-frozen in liquid nitrogen and then total RNA was prepared from homogenized specimens using TRIzol reagent (Invitrogen, Carlsbad, CA, USA). Extracted RNA was quantified by measuring absorbance at 260 nm with a Genova UV/visible spectrophotometer. First-strand cDNAs were prepared from the total RNA specimens as described above. After reverse transcription, all real time PCR reactions were performed on the Bio-Rad MiniOpticon™ Real-Time PCR System using SYBR Premix Ex Taq™ (TaKaRa, Shiga, Japan) with gene-specific primers to amplify ArSLK and tubulin as an internal control (ArSLK–3′F3 and ArSLK–RTR for ArSLK; Tubulin–F and Tubulin–R for tubulin) (Table 1). The relative mRNA amounts were analysed by the comparative CT method as described by Schmittgen and Livak [23]. All data were expressed as means + SE from three independent repetitions. All statistical analyses were performed by a two-tailed, paired Student's t test, and the differences were considered significant for P<0.01.

### Stress treatments

The post-diapause cysts were rehydrated and hatched as described above. The fresh hatched nauplius larvae, which are incubated at 25°C, were used for the following stress treatments. For heat shock, nauplius larvae were directly exposed in 28 g/L artificial seawater at 42°C in a water bath for 30 min and 60 min. For acidic pH stress, larvae were cultured in 28 g/L artificial seawater at pH 3.0 for 30 min and 60 min. For UV irradiation, nauplius larvae were exposed directly to a UV lamp (310 nm) at 0.15 joules/cm^2^ for 15 min and 30 min according to the previous study [24].

Larvae collected after exposure to heat shock (60 min), acidic pH exposure (60 min), and UV irradiation (30 min) were incubated in 28 g/L artificial seawater for 7 h under laboratory conditions. About 90 percent nauplius larvae resumed their abilities of free-swimming and the live larvae were obtained as the stress released specimens.

### RNA interference (RNAi)

For double-strand RNA (dsRNA) preparation, plasmid PET-T7 that contained two inverted T7 polymerase sites flanking the cloning region was used as the dsRNA expression vector [25]. To obtain the reconstructed plasmid expressing the dsRNA of *ArSLK*, a 580 bp fragment in the coding region of *ArSLK* gene was amplified with specific primers (ArSLK–dsF and ArSLK–dsR) (Table 1), excised and subcloned into PET-T7 at XbaI and EcoRI sites. The recombinant plasmid transformed into *E. coli* DH5α firstly for confirming the inserted nucleotide sequence by DNA sequencing, and then into *E. coli* HT115 for expressing dsRNA. The plasmid expressing dsRNA targeting GFP had been constructed as previously described and used as the negative control [26]. The dsRNA was produced and purified according to the methods reported by Yodmuang *et al* [27].

*Artemia* adults were injected with 1 μg dsRNA targeting *ArSLK* or *GFP*, respectively, using the Ultra-MicroPump II equipped with the Micro4TM MicroSyringe pump controller (World Precision Instruments, Sarasota, FL, USA). After injection, animals were cultured in 55 g/L artificial seawater with 16 h light per day. Eight ovisac specimens were isolated from adults at the early embryo stage according to the method of Liu *et al.* [26] to extract RNA and protein, followed by RT-PCR and Western blotting for assessing the RNAi efficiency. Three independent groups were repeated for analysis.

### Analysis of the stress resistance of larvae after RNAi-treatment

Nauplius larvae, released by *ArSLK* or *GFP* RNAi-treated *Artemia* adults, were collected for stress treatment as described above. Fifty larvae were used for each group. The number of free-swimming nauplius larvae was recorded every 5 min in the heat shock, acidic pH and UV irradiation stress groups.

All data are given as means + SE from three independent repetitions. All statistical analyses were performed by a two-tailed, paired Student's *t* test, and the differences were considered significant for *P*<0.01.

## Results

### Identification and characterization of *ArSLK* cDNA

**Figure 1:**
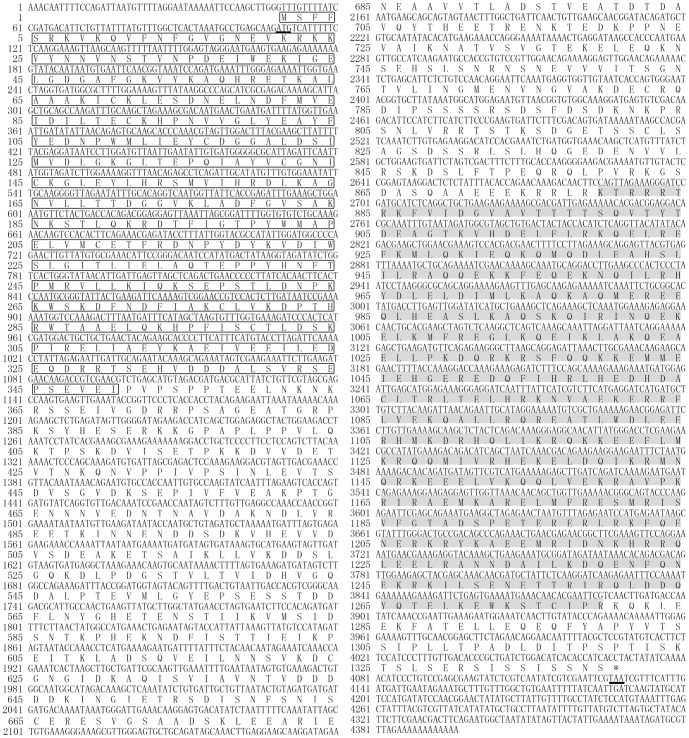
The nucleotide sequence and deduced amino acid sequence of ArSLK. The start and stop codons are indicated by bold and underlined text. The conserved N-terminal kinase domain (residues 1–349) is boxed. The coiled-coil domain at the C-terminus (residues 879–1279) is indicated by grey highlighting.

The full-length cDNA encoding *ArSLK* was isolated from *Artemia parthenogenetica*. A sequence analysis revealed that the 4396 bp cDNA encodes a 1340 amino acid protein with a predicted molecular mass of 153.1 kD (Fig. 1).

**Figure 2:**
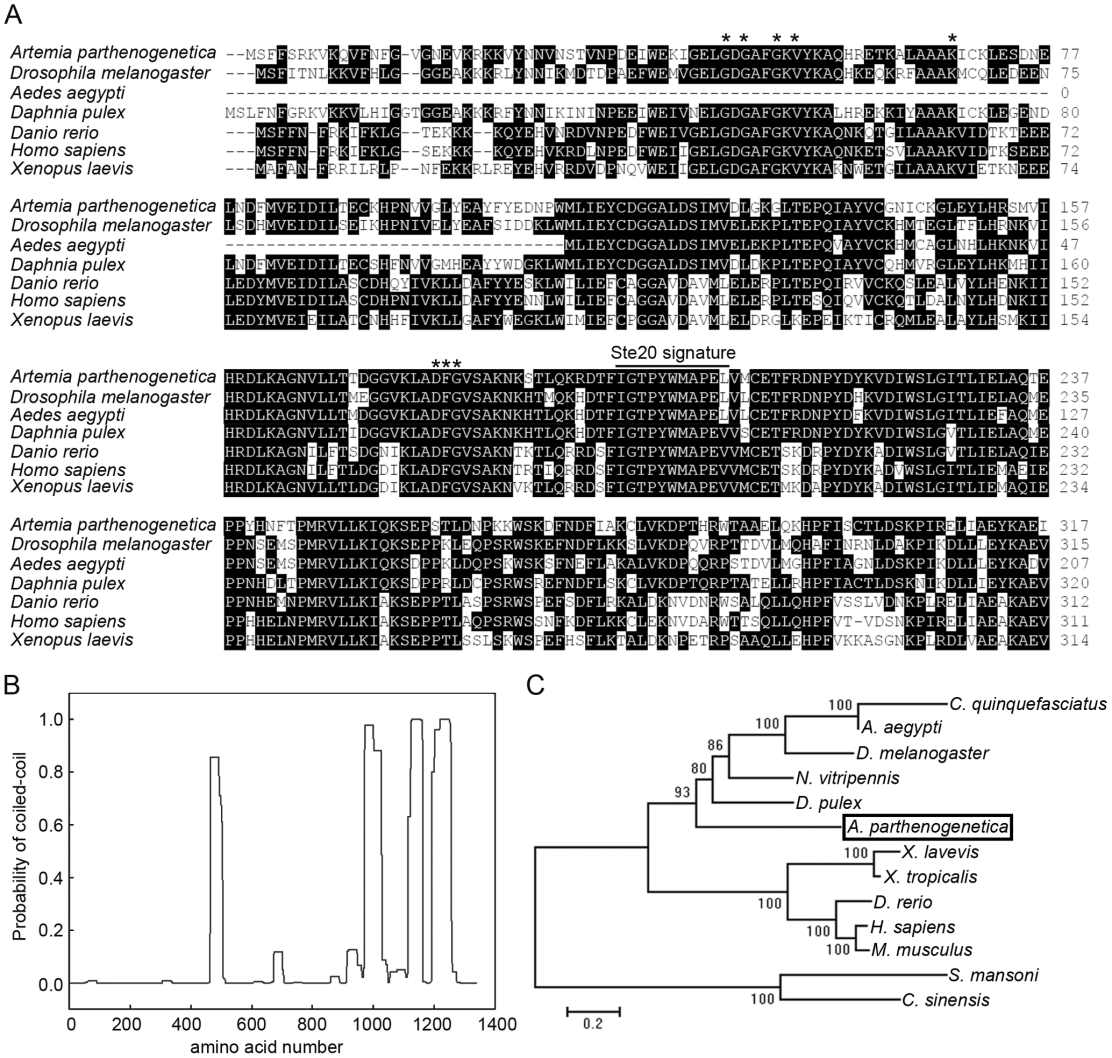
Sequence analysis of ArSLK. (**A**) Alignment of the amino acid sequence of the N-terminal kinase domain of ArSLK with those of SLKs from other species. The Ste20 signature sequence is highlighted and conserved amino acid residues are indicated by asterisks. The black shaded areas represent identical amino acid residues. (**B**) Analysis of the coiled-coil region of the ArSLK. The probability of formation of a coiled-coil structure was calculated for each residue with a window of 28 amino acids using the ‘COILS’ program. (**C**) Phylogenetic analysis of the amino acid sequences of SLKs from multiple species. GenBank accession numbers of the sequences used in the amino acid alignment and phylogenetic analysis are as follows: *Artemia parthenogenetica*, KC818632; *Drosophila melanogaster*, NP_726441; *Aedes aegypti*, XP_001649074; *Daphnia pulex*, EFX76394; *Danio rerio*, NP_001139073; *Homo sapiens*, NP_055535; *Xenopus laevis*, NP_001079164. *Cluex quinquefasciatus*, XP_001864707; *Nasonia vitripennis*, XP_001603525; *Xenopus troplicalis*, NP_001072623; *Mus musculus*, NP_033315; *Schistosoma mansoni*, AAN72832; *Clonorchis sinensis*, GAA30009.

Alignment of the N-terminal amino acid sequence of ArSLK revealed that the kinase domain is conserved across multiple species (Fig. 2A). The consensus sequence G46-X-G48-X-X-G51, which is essential for ATP binding, and the adjacent V53 residue, which is necessary for the correct positioning and interaction of the conserved glycine residues with ATP, were identified. A conserved lysine residue within the ATP-binding site was identified at position 68, and the Ste20 signature motif (IGTPYWMAPEV), characteristic of SLK family members [14], was also identified at residues 196-207 (Fig. 2A).

The structure of ArSLK was predicted using the ‘COILS’ program [28]. The result revealed that the C-terminal region of the protein, spanning residues 880-1280, has a high probability of forming a coiled-coil conformation (Fig. 2B). A phylogenetic analysis, performed using the method of Hanks and Hunter [29], revealed that ArSLK is in the crustacean clade and is closely related to SLK from *Daphnia pulex* (Fig. 2C).

### Expression of ArSLK in *Artemia* during development

**Figure 3:**
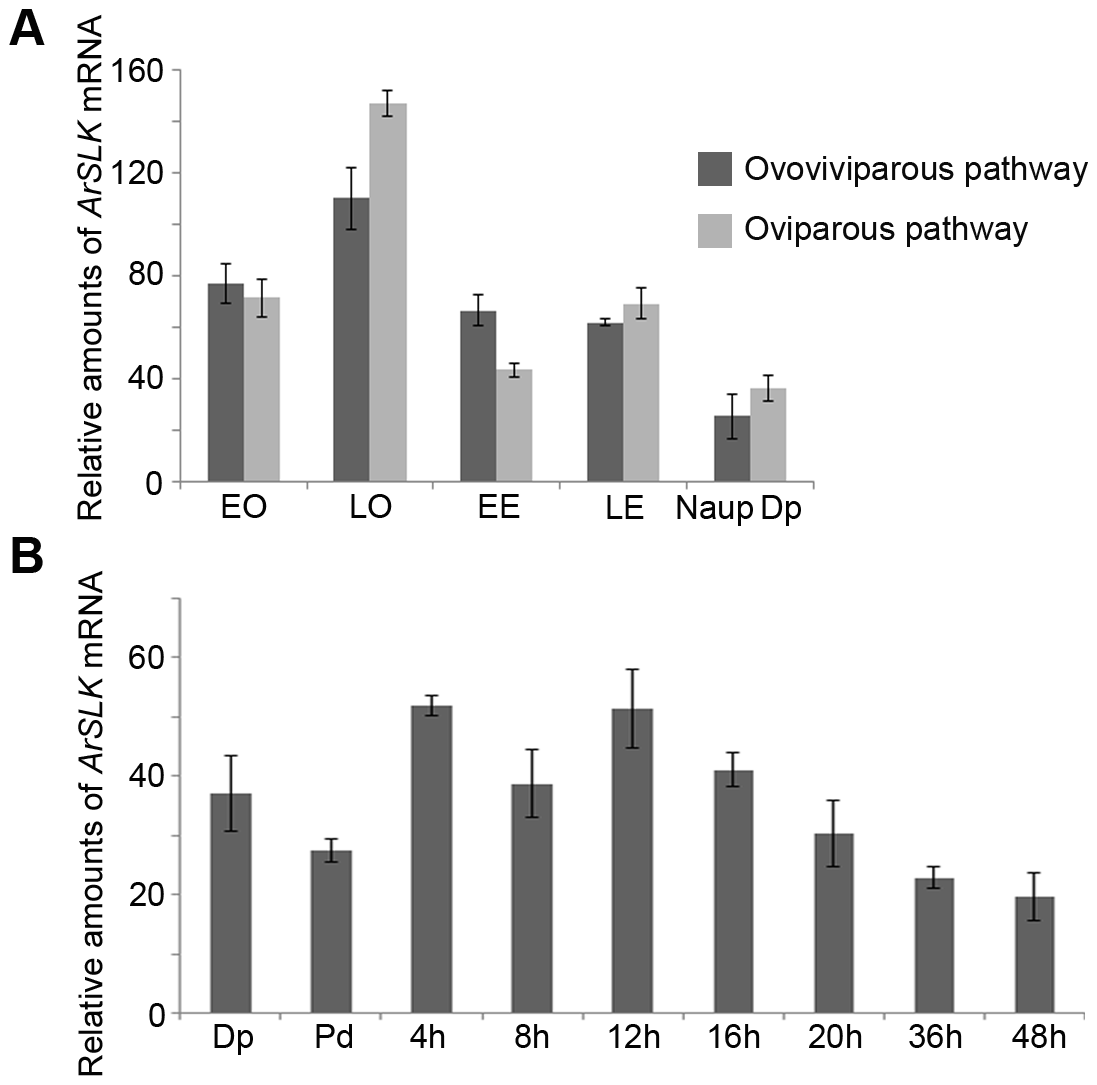
The relative amounts of *ArSLK* mRNA detected by real-time PCR. The mRNA amounts were normalized to those of *tubulin* mRNA. (**A**) The relative amounts of *ArSLK* mRNA in oocytes and embryos of ovoviviparous and oviparous *Artemia*. EO, early oocytes; LO, late oocytes; EE, early embryos; LE, late embryos; Naup, nauplius larvae; Dp, diapause cysts. (**B**) The relative amounts of *ArSLK* mRNA during the hatching process of *Artemia*. Dp, diapause cysts; Pd, post-diapause cysts. The times (h) represent the incubation times of the post-diapause embryos. (**A, B**) Data are represented as mean + SE of n = 3 independent repeats. Statistical analyses of the differences between expression levels in the two pathways were performed by two-tailed, paired Student's *t* tests. No significant differences (*P*<0.05) were detected.

Real-time PCR was used to analyze the amounts of the *ArSLK* mRNA. There were no significant differences between *ArSLK* mRNA amounts in any stage of the two developmental pathways (Fig. 3A). But amounts of *ArSLK* mRNA were higher in the late oocyte stage (LO) than in all other stages of both pathways (Fig. 3A). Amounts of *ArSLK* mRNA were highest during earlier development (4–16 h) compared to later periods (20–48 h) (Fig. 3B).

**Figure 4:**
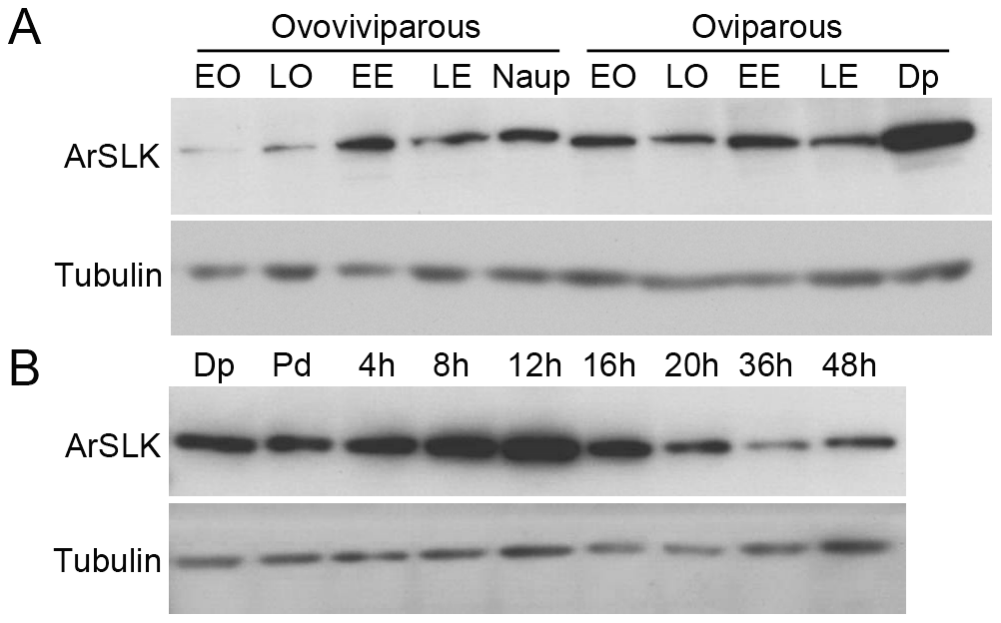
Immunoblot of amounts of ArSLK protein during development. Tubulin was measured as a loading control. The developmental stages are labelled as described in the legend for Figure 3. (**A**) ArSLK protein in oocytes and embryos of ovoviviparous and oviparous pathways. (**B**) Amounts of ArSLK protein during development of post-diapause cysts.

Immunoblotting was used to estimate the amounts of ArSLK protein, which were very low in oocytes of the ovoviviparous pathway with modest increases in later stages (Fig.4A). The amounts of ArSLK were higher in the oviparous pathway with a dramatic increase in diapause cysts (Fig. 4A). Amounts of ArSLK protein remained about the same during the initial 4 h of development of post-diapause cysts, but then increased substantially, peaking at about 12 h then falling to much lower levels at the later developmental times (Fig. 4B).

### Subcellular location of ArSLK during *Artemia* development process

**Figure 5:**
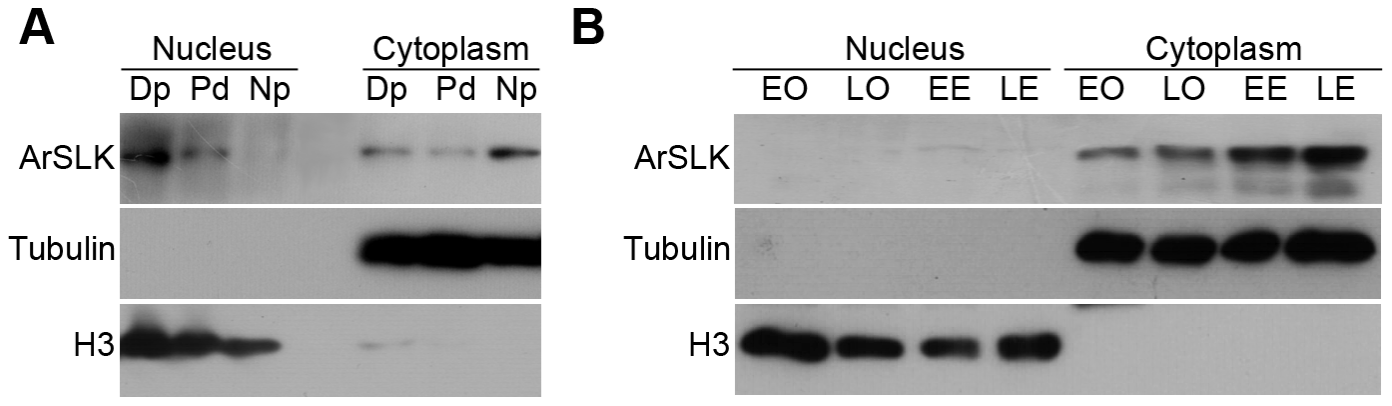
Immunoblot analysis of the subcellular location of ArSLK during development. Cell fractionation was performed and tubulin and H3 were used as markers of the cytoplasmic and nuclear fractions, respectively. (**A**) Dp, diapause cysts; Pd, post-diapause cysts; Np, nauplius larvae. (**B**) Nauplius-destined oocyte and embryonic developmental processes of *Artemia*. EO, early oocytes; LO, late oocytes; EE, early embryos; LE, late embryos.

The subcellular distribution of proteins is often related to their specific functions. To examine the subcellular location of ArSLK, cell fractionation was performed as described in the methods section. Successful fractionation was confirmed using anti-α-tubulin and anti-histone H3 antibodies as cytoplasmic and nuclear markers, respectively (Fig. 5A and 5B). The results revealed that ArSLK was detected mainly in the nuclear fractions from diapause and post-diapause embryos, and in the cytoplasmic fraction from nauplius larvae (Fig. 5A), while maintained in the cytoplasm during the whole ovoviviparous developmental process (from oocytes to embryos) (Fig. 5B).

### The nuclear localization of ArSLK is required for the regulation of the stress response

**Figure 6:**
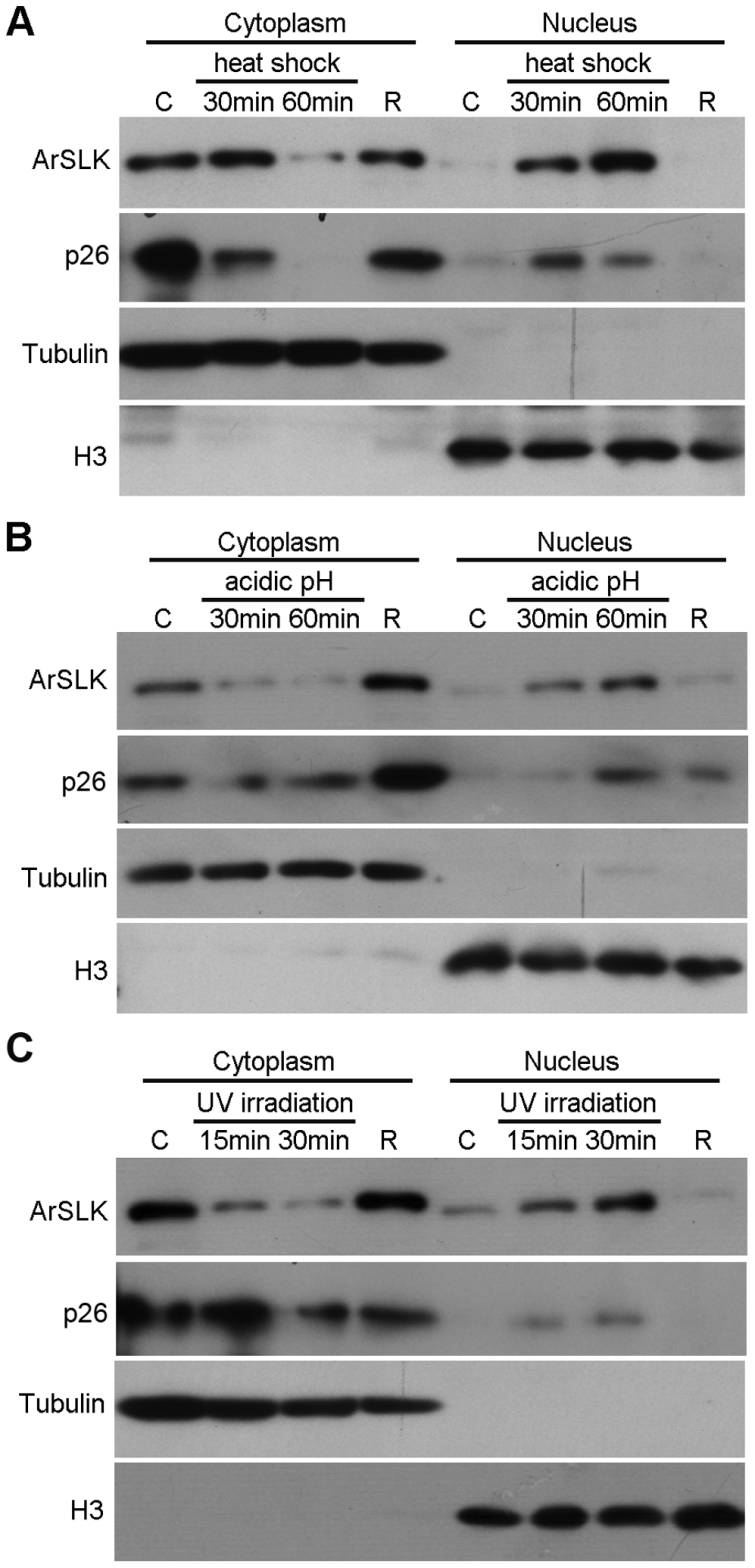
Immunoblot analysis of the subcellular locations of ArSLK and p26 after exposure to 42°C heat shock (A), acidic pH stress (B), and UV irradiation (C). Tubulin and H3 were used as markers of the cytoplasmic and nuclear fractions, respectively. C, control group with no stress treatment; R, nauplius larvae after stress that underwent subsequent stress-free incubation for 7 h.

Since ArSLK was differentially localized during *Artemia* development, experiments were performed to determine whether nuclear ArSLK is important to stress resistance. A cell fractionation assay was used to examine the location of ArSLK after exposure of nauplius larvae to the following stress treatments: heat shock (42°C) for 30 min and 60 min, acidic pH (pH 3.0) for 30 min and 60 min, UV irradiation (0.15 joules/cm^2^) for 15 min and 30 min. The results show that the ArSLK protein translocated from cytoplasm to nucleus after exposure to stresses. Moreover, as the exposure time extended, almost all the ArSLK translocated into the nucleus (Fig. 6A, 6B and 6C).

Following the treatments, a stress-release process was performed (R in all parts of Fig. 6) in which nauplius larvae were transferred to normal environmental culture conditions. Cell fractionation analysis revealed that the levels of ArSLK protein in the nuclear and cytoplasmic fractions of all stress-released larvae were similar to those of the unstressed control groups (C in all parts of Fig. 6).

The subcellular location of p26 in the both processes mentioned above was also observed. Cell fractionation analysis revealed that p26 was distributed mainly in the cytoplasm of control nauplius (C in all parts of Fig. 6), and then p26 in the nucleus increased during the stress process (Fig. 6A, 6B and 6C). It returned to the cytoplasm after stress released (R in all parts of Fig. 6).

### Decrease in *ArSLK* reduces the stress tolerance of nauplius larvae

**Figure 7:**
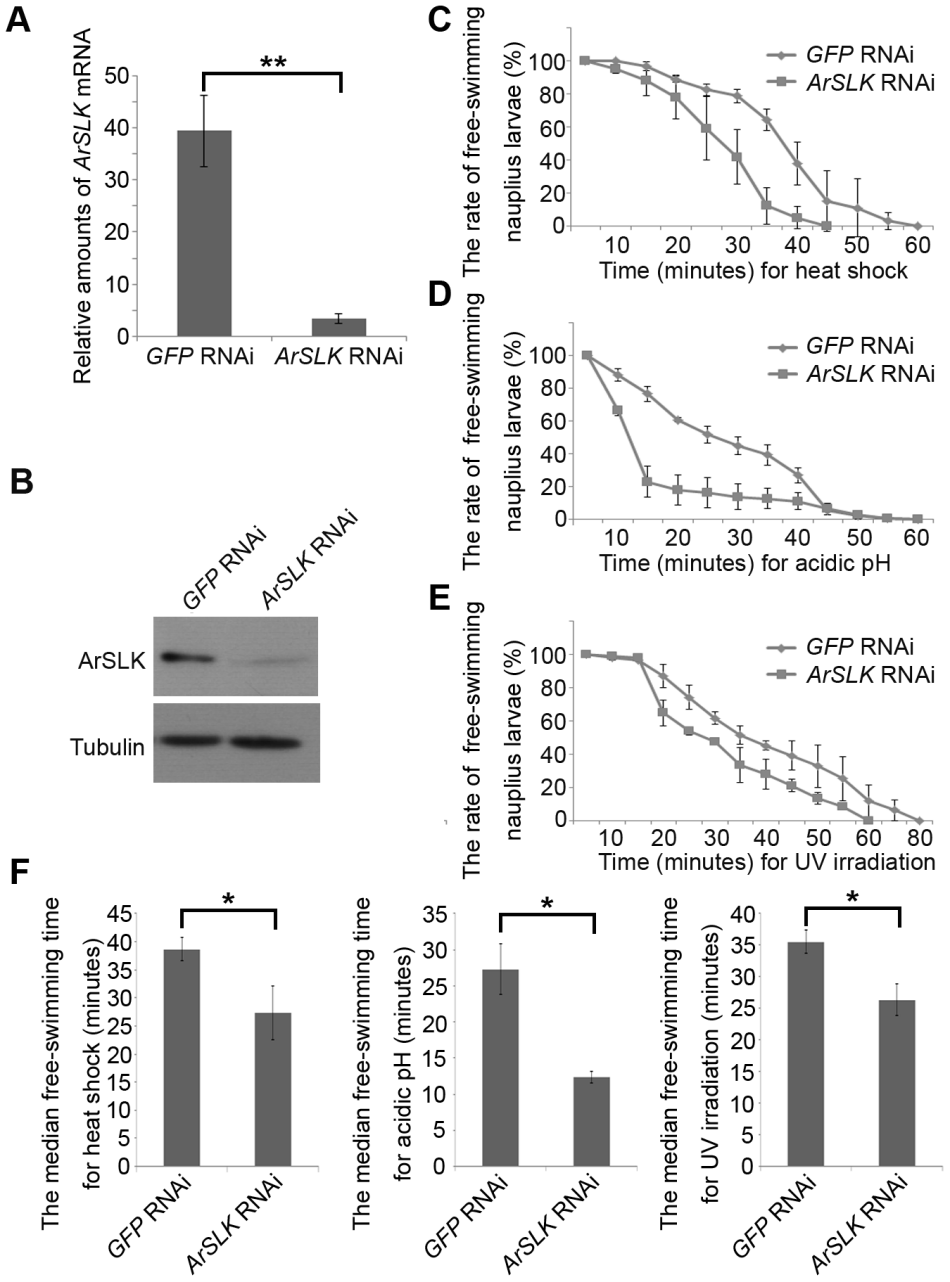
The effects of knockdown of the ArSLK gene on nauplius stress tolerance. (**A**) Real-time PCR analysis of relative amounts of ArSLK mRNA in Artemia adults treated with GFP-specific or ArSLK-specific RNAi. The mRNA amounts were normalized to those of tubulin mRNA. (**B**) Immunoblot analysis of ArSLK protein in the two RNAi-treated groups. Tubulin was used as a loading control. (**C–E**) The rates curves of free-swimming nauplius larvae after 42°C heat shock (**C**), acidic pH stress (**D**), and UV irradiation (**E**). (**F**) The median free-swimming times of nauplius larvae from RNAi-treated adults under three mentioned stresses were exhibited by histograms. Data are represented as mean + SE of n = 3 independent repeats. Statistical analyses of the differences between the GFP-RNAi and ArSLK-RNAi groups were performed by two-tailed, paired Student's t tests. ** P<0.01.

To address further the function of ArSLK during stress resistance, gene knockdown by RNAi with *Artemia* adults was performed. The efficiency of knockdown was evaluated by the females at the early embryo stage using real-time PCR (Fig. 7A) and immunoblot analyses (Fig. 7B). The outcome showed that the amounts of endogenous ArSLK, i.e., both mRNA and protein in the *ArSLK*-specific RNAi group, were significantly lower than those in the control *GFP* RNAi group (Fig. 7A and 7B).

Nauplius larvae released from RNAi-treated adults were collected to examine their ability to resistant environmental stress. The swimming performance of animals as the stress survival response was observed [30]. Both the recorded rate curve of free-swimming nauplius larvae and the median free-swimming time were employed to evaluate stress tolerance ability. The results revealed that, following all stress treatments, including heat shock, acidic pH and UV irradiation, the nauplius larvae released from females receiving the *ArSLK* dsRNA lost the ability of free-swimming much earlier than those of control larvae released from adults injected with *GFP* dsRNA (Fig. 7C, 7D, 7E and 7F). Overall, these data provide evidence that a decrease in *ArSLK* reduces stress tolerance of nauplius larvae.

## Discussion

It has been very well documented that *Artemia* have an outstanding ability to withstand extremely adverse condition [1], [2]. However, the intracellular signal transduction mechanisms that respond to environmental stresses are largely unknown. In the present study a homolog of mammalian SLK, called *ArSLK*, was identified and its role in stress resistance of *Artemia* was investigated.

Homologs of SLK have previously been identified in various species. SLK family members contain characteristic conserved N-terminal kinase domains and variable C-terminal non-catalytic domains [14], both of which were identified in ArSLK (Figs. 1 and 2). The coiled-coil structure in the C-terminal region can mediate protein-protein interactions and contribute to enhancing the kinase activity of human SLK via homodimerization [28], [31]. Therefore, we speculate that ArSLK might also possess a kinase activity that is regulated in the same manner as that of human SLK.

*Artemia* diapause cysts with programmed metabolic and developmental arrest were formed and released under extremely stressful conditions. It is precisely because of this arrest state helping to ensure survival of this species in stress resistance [32]. In this study, we investigated the subcellular distribution of ArSLK in diapause embryos and the nauplius-destined developmental process. We found the nuclear ArSLK accumulated in diapause embryos (Fig. 5A), in which cell division was restricted and development was arrested at the gastrulae stage. In the nauplius and nauplius-destined embryos with cell division and continued embryonic development, ArSLK was mainly distributed in the cytoplasmic fraction (Fig. 5A and B). Thus, we proposed that the accumulation of ArSLK in the nucleus plays a role in developmental arrest to resistant environmental stress in diapause cysts.

Trafficking of an activated signal component or its substrates from the cytoplasmic to nuclear compartment is required for the transduction of extracellular signals. Studies in rabbit brain showed that the stress-induced expression and nuclear translocation of hsp70 are characteristic features of the response to heat shock [33]. A previous study in *Artemia* also found that p26, a small heat shock protein, reversibly moves to the nucleus in response to stresses such as heat shock, acidic pH and anoxia during early development of activated (post-diapause) cysts [34]. To prove the role of ArSLK in stress resistance, the stress treatment assay of nauplius was performed. After stress-treated, both p26 and ArSLK in the nucleus were increased, while they translocated to the cytoplasm after stress releasing. Moreover, the result of gene knockdown revealed that the nauplius larvae from *ArSLK* RNAi group lost the ability of free-swimming much earlier than those from the *GFP* RNAi group when suffered the same stress. Based on these results, we suggested that nuclear translocation of ArSLK may also be a response mechanism to involve in the stress resistance via cell cycle regulation to development. However, the molecular mechanisms involved in this resistance are not well understood.

Previous studies have been proved that there are a number of links between the stress-response and cell cycle checkpoint pathway [35]. SLK was reported to play a role in cell proliferation. This regulation is achieved by phosphorylating Plk1 [20].Although the *in vitro* activity of SLK phosphorylated on Plk1 has been proved in the previous studies [19], [20], there is still controversy regarding the relationship between these two kinases *in vivo*. The variations in the cytoplasmic ArSLK levels during development described in our present study (Fig. 5) parallel the levels of Plk1 activation described previously [36]. For example, cytoplasmic ArSLK and activated Plk1 levels were both high in nauplius larvae and lower in diapause cysts [36], suggesting that cytoplasmic levels of ArSLK may be correlated with the level of Plk1 activation. However, the results of RNAi-mediated knockdown of *Plk1* and *ArSLK* were confusing. Pseudo-diapause cysts were released following knockdown of *Plk1* [36], while nauplius larvae were produced after knockdown of *ArSLK*. Therefore, ArSLK may not be the only kinase responsible for phosphorylating Plk1. Similar result has been obtained in the *Drosophila* S2 cells, in which the depleted dPlkk has not impaired the activation of Plk1 [37]. The limited phosphorylation of Plk1 in the diapause cysts also might be correlated with the massive drop of ATP concentration for the global reduced metabolic activity, which induced the arrest of the enzymatic activity.

The stress kinases p38MAPK and JNK have also been reported to be the downstream of SLK signalling pathway and play a role in the linkage of stress and cell cycle checkpoint [17], [18], [35]. Thus, we speculate that p38MAPK and JNK mediate the regulation of ArSLK in stress resistance via cell cycle and developmental arrest.

In conclusion, the results of this study showed that ArSLK is translocated to the nucleus in both diapause cysts and also in nauplius larvae following exposure to environmental stresses. In addition we found that this translocation was involved in the regulation of stress tolerance in nauplii. We suggest that the trafficking of ArSLK is also involved in the stress resistance of diapause cysts via regulating developmental arrest, but we accept that more evidence is needed in that regard. These findings shed light on the functions of members of the SLK family and may provide a new regulatory strategy for stress responses.
